# Intestinal Barrier Permeability in Allergic Diseases

**DOI:** 10.3390/nu14091893

**Published:** 2022-04-30

**Authors:** Monika Niewiem, Urszula Grzybowska-Chlebowczyk

**Affiliations:** Department of Pediatrics, Faculty of Medical Sciences, Medical University of Silesia in Katowice, 40-752 Katowice, Poland; urszulachlebowczyk@wp.pl

**Keywords:** food allergy, children, tight junctions, dysbiosis, microbiome

## Abstract

The role of intestinal permeability (IP) markers among children and adults with food allergies is not fully understood, and the identification of biological indicators/markers that predict growth retardation in children with allergic diseases and atopy has not been well explained. Studies have shown that patients with atopic diseases respond abnormally to food allergens. Accordingly, differences in the types of immune complexes formed in response to antigen challenges are significant, which seems to underlie the systemic signs of the food allergy. Increased intestinal permeability over the course of a food allergy allows allergens to penetrate through the intestinal barrier and stimulate the submucosal immune system. Additionally, the release of cytokines and inflammatory mediators enhances the degradation of the epithelial barrier and leads to an improper cycle, resulting in increased intestinal permeability. Several studies have also demonstrated increased permeability of the epithelial cells in those afflicted with atopic eczema and bronchial asthma. Ongoing research is aimed at finding various indicators to assess IP in patients with atopic diseases.

## 1. Introduction

A considerable increase in the incidence of allergic diseases has been observed in recent years. They constitute one of the most serious health issues in developed countries and affect both the pediatric and adult populations. Data show that the proportion of patients with atopic diseases is as high as 40%, and this is a growing trend [1]. The cause of increasing allergy cases are mostly environmental factors, which have a significant impact on the composition of gastrointestinal microorganisms. Currently, the intestinal microbiota is considered to be the largest and most active component of the intestinal barrier and is necessary for the optimal development of immune tolerance and the function of the immune system. The intestinal microbiome develops for approximately the first 1000 days of life, including the prenatal period and about 2 years after birth. Therefore, factors that influence the development of the microbiome and reduce the risk of allergy are strongly emphasized: natural birth, breastfeeding, contact with nature, having pets, appropriate diets (e.g., high-fiber food, fermented products, home-made food), as well as the consumption of probiotics and prebiotics [2,3]. They have a strong effect on the development of the gastrointestinal microbiome, e.g., on the condition of intestinal mucosa and the programming of the child’s immature immune system [3].

Normal functioning of the intestinal mucosa is very important for health because it is a physiological functional unit that separates the intestinal lumen from the inner environment of the body and performs protective, nutritional and immune functions [4]. The intestinal mucosa is mainly responsible for maintaining the balance between the absorption of nutrients and ions, fluid secretion, protection from microorganisms, as well as food toxins and antigens in the intestinal lumen. Due to its complexity, the intestinal mucosa is highly sensitive to environmental and alimentary factors. It becomes damaged in the case of overexposure, leading to increased intestinal permeability. Compromised functioning of the intercellular junctions in the intestinal wall results in a complete or partial loss of control over the agents that penetrate the bloodstream [3,4].

## 2. Intestinal Barrier

### 2.1. Characteristics of the Intestinal Barrier Structure

The intestinal barrier is a physiological functional unit that separates the intestinal lumen from the inner environment of the body. It is composed of a mucus layer that contains microorganisms present in the intestinal lumen, intestinal epithelium and the cells of the blood, lymphoid, immune and nervous systems [4].

### 2.2. Mucus Layer

The mucus layer is the first line of defense for the intestinal barrier. It prevents various microorganisms from adhering to and penetrating through the intestinal wall. It consists of an inner and outer layer [4].

### 2.3. Outer Mucus Layer

The outer mucus layer of the intestinal barrier is rich in antibacterial peptides (synthesized by the Paneth cells) and immunoglobulin A (produced by the plasma cells). It is also the natural habitat of many microorganisms. These are the largest and the most active components of the intestinal barrier. The microbiota is a group of microorganisms that colonize the human body. The intestinal microbiota consists of all the microorganisms that colonize the intestines. Accordingly, the more general term of gastrointestinal microbiota is applied. In addition, there are skin and respiratory microbiotas. Among all of them, the intestinal microbiota is the most abundant and diverse [4,5,6].

### 2.4. The Intestinal Microbiome

The outer mucus layer of the intestinal barrier is a specific microbiological niche that creates one of the most dynamic ecosystems containing the most diverse species. It changes throughout human life and constantly aims to reach a state of equilibrium. The microbiota of a healthy human body basically consists of anaerobic bacteria and (additionally, but in smaller amounts) aerobic bacteria, viruses and fungi [4]. Particular parts of the intestine are colonized by specific populations of microorganisms that compete for the best environmental conditions and nutrients (which sometimes contain pathogens). Thus, they are important protective agents as they competitively inhibit the overgrowth of other microorganisms that are harmful to humans [5,7].

There are over 1500 species of microorganisms in the intestine and their total weight may reach 1.5 to even 2 kg [6]. We cannot precisely determine the optimal types and amounts of bacteria which should be present in the human intestine as no standards have been established. The composition of the intestinal microbiota is strictly individual and is characterized by large population differences. Interestingly, it has been suggested that the presence of certain bacterial species may predispose patients to develop certain diseases such as allergies, obesity, inflammatory bowel disease or cancer [8,9,10].

In addition to natural birth, breastfeeding, contact with nature, having pets, following appropriate diets (e.g., high-fiber food, fermented products, home-made food) and the consumption of probiotics and prebiotics, the optimal microbiome is influenced by the environment of the developing young person. It is known that the intestinal microbiome is less diverse in single children compared to children who have siblings [11,12]. Furthermore, contact with animals is an important factor that has a beneficial effect on the development of required microbiota [13]. Certain studies show that children who live in rural environments, where daily contact with animals is typical, the living conditions are not as sterile as in urban areas and hygiene regimes are not so strict, present more diverse microbiomes which are more beneficial for their health [4,11,12,13,14]. Antibiotic treatments have negative effects on the development of the microbiome [15]. Increasingly, there are more reports suggesting that non-steroid anti-inflammatory drugs and proton-pump inhibitors may also affect the microbiome [16,17].

The composition of the intestinal microbiota also depends on the methods of feeding during infancy and early childhood. Human milk contains three groups of agents that modulate the composition of the intestinal microbiome: prebiotic oligosaccharides, probiotic living bacteria [18,19] and postbiotics [20]. Postbiotics are products of bacterial metabolism or components of bacterial cell degradation. They demonstrate health-promoting effects on the human body [20].

There are many studies concerning probiotic oligosaccharides and their impact on the development of the intestinal microbiome. A special emphasis is placed on mixed short-chain galactooligosaccharides (GOS) and long-chain fructooligosaccharides (FOS) in a 9:1 proportion. Many papers reveal that the use of the above agents in appropriate proportions in artificial milk products induce and change the profile of infants’ intestinal microbiomes. These agents aim to achieve the profile characteristics of those observed in breastfed children (increased amounts of the required *Lactobacillus* and *Bifidobacterium* species have been reported). Moreover, the added product has been observed to benefit the restoration of microbiological balance following antibiotic treatment [21,22]. Therefore, supplementation of GOS/FOS in a 9:1 proportion supports immune system function and reduces the number of infections and allergic diseases (AD, urticaria or wheezing) [23,24,25].

The intestinal microbiota performs many important functions in the human body, including protective, metabolic, trophic and immune functions [4]. Immune function is particularly important when considering antigen elimination. The intestinal bacteria stimulate the production of mucins, i.e., glycoproteins which protect the intestinal epithelium from the invasion of microorganisms and toxins, so they protect the intestinal epithelium from harmful colonization and the growth of pathogenic bacteria [26]. Therefore, microorganisms in the gastrointestinal system create the first line of defense in the body. They seal the intestinal barrier, and improve immune tolerance and processes that control the response to potentially harmful allergens that invade the body. The colonies of bacteria in the gastrointestinal system are the first to have contact with a child’s immature immune system and they stimulate the lymphocytes that regulate cytokine balance, i.e., the Treg cells. Moreover, the intestinal bacteria are responsible for the activation of B cells to synthesize antibodies, mainly the secretory antibodies (such as immunoglobulin A). The intestinal bacteria also express proteins (zonulin, occludin) that co-create and modulate the work of interepithelial junctions [26,27].

The efficient and rapid elimination of antigens is a molecular process that involves the connection of toll-like receptors (TLRs) located on the intestinal epithelium, on dendritic cell projections and nucleotide oligomerization domains (NODs) with the structures present on bacterial cells that trigger the secretion of inflammatory mediators [4,28]. The contact with antigens results in the stimulation of the signaling pathway that activates the effector cells of the immune system, including macrophages, NK cells, B cells, helper T cells (Th1 and Th2), cytotoxic T cells and Treg lymphocytes. The Treg lymphocytes regulate the immune system response and are responsible for the production of interleukin 10 (IL-10), as well as the synthesis of the transforming growth factor β1 (TGF-β1). In addition, they maintain the Th1/Th2 cytokine homeostasis and control the development of immune tolerance in the body [29]. This is particularly important during early childhood, when the ability of Th1 lymphocytes to produce cytokines (IL-12, IFN-gamma) is compromised and the cytokine profile of the T cells is initially directed at the production of proallergic Th2 lymphocytes [30].

Current reports suggest that the intestinal microbiome is less diverse in children with food allergies. In these patients, fewer *Bacteroidetes*, *Bifidobacterium* and *Lactobacillus* colonies have been observed [31,32]. Importantly, differences in the composition of microbiota between healthy children and patients with atopic diseases are observed during early infancy, before the clinical manifestations of the allergy appear. This has been confirmed by Kalliomaki et al. In their research, they discovered that children with allergies have smaller amounts of the *Bifidobacterium* species and larger amounts of *Clostridium* bacteria compared to healthy children without the signs and symptoms of allergic disease [33]. Moreover, it has been observed that a less diverse intestinal microbiome during the first year of life is associated with the development of asthma at seven years of age [30,32]. The studies by Sjögren et al. also show that smaller amounts of *Bifidobacterium* and *Lactobacillus* species may lead to the development of allergies in five-year-old children. Furthermore, children with allergies are poorly colonized by lactic acid bacteria during early infancy (*Lactobacillus rhamnosus, Lactobacillus casei, Lactobacillus paracasei* and *Bifidobacterium adolescentis* species, in particular) during early infancy. In their reports, the researchers emphasize the fact that colonization of the digestive tract by *Bifidobacterium* and *Lactobacillus* species brings about protection from allergies, and they suggest that colonization by *Clostridium difficile* bacteria may be associated with the risk of allergy development [30].

In summary, having a more diverse intestinal microflora during the early stages of life may prevent the development of allergies.

### 2.5. Inner Mucus Layer

The inner mucus layer is thicker than the outer layer and directly adheres to the neighboring epithelial cells. This layer is responsible for the hydration of epithelial cells, the control of regeneration processes and protection from digestive enzymes. The inner layer mainly consists of glycocalyx, i.e., the carbohydrate layer produced by the goblet cells. It limits the penetration of antigens into the lamina propria of the intestinal mucosa [4,34].

### 2.6. Cells of the Blood, Lymphoid, Immune and Nervous Systems

The lamina propria of the mucosa is located under the single layer of epithelial cells. It forms the intestinal villous stroma and separates the intestinal crypts. The lamina propria of the mucosa contains a very abundant network of blood and lymph vessels, as well as nerve fibres. The gastrointestinal system contains mucosa-associated lymphoid tissue (MALT), a part of which is formed by gut-associated lymphoid tissue (GALT). The GALT cells are the sites where the immune response is induced and they may be dispersed within the intestinal epithelium (intraepithelial CD8+ T cells, in addition to plasma cells, eosinophils, macrophages, mast cells and dendritic cells). Furthermore, they occur as organized lymphoid tissue and form lymphoid follicles, Peyer’s patches and mesenteric lymph nodes [35].

## 3. Intestinal Epithelium and the Structure of Tight Junctions

The most important component of the intestinal barrier is a single layer of epithelial cells formed by enterocytes. These constitute 80% of the layer [34]. The epithelial layer is mainly responsible for the process of nutrient absorption. Thus, it influences the development of immune system activity, and controls the release of cytokines and the expression of receptors involved in the immune system response. The following cells are placed between the epithelial cells: goblet cells, enterochromatophilic cells, Paneth cells and M cells. The goblet cells secrete mucus, the enterochromatophilic cells release hormones and neuropeptides, the Paneth cells synthesize defensins and the M cells capture antigens from the intestinal lumen [36].

**Figure 1:**
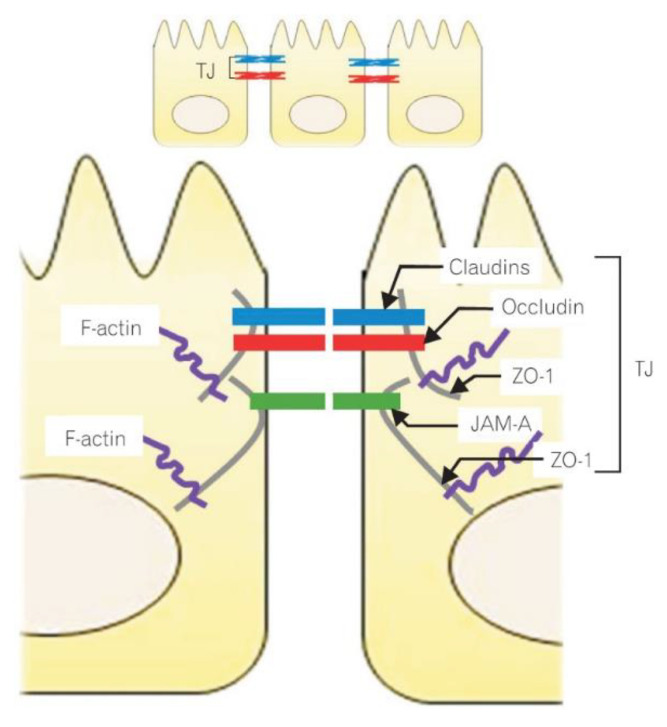
Molecular structure of the intracellular junction of intestinal epithelial cells. Reprinted with permission from Ref. [37]. 2015, Lee, S.H. ZO-1-zonula occludens 1; JAM-A-junctional adhesion molecule A; TJ-tight junctions.

The epithelial cells are held together by tight junctions (TJs), adherens junctions and gap junctions. The key component controlling intestinal barrier permeability are TJs, which were first described in the 1970s by Farquhar and Paladeand. They are located on the upper lateral surface of the cell membrane [37]. The TJs are the most important structures responsible for the integrity and selectivity of the permeable epithelial layer, e.g., they control the passive transport of water-soluble particles. The TJs are multiprotein complexes formed by four types of transmembrane proteins: claudins, occludins, junctional adhesion molecules (JAMs) and tricellulin [38]. Their intracellular domains interact with one another and with the proteins of the zonula occludens (ZO) (i.e., the cytosolic proteins, including ZO-1, ZO-2 and ZO-3), that connect with actin filaments (components of the enterocyte cytoskeleton). The interaction of occludins, claudins, JAMs and tricellulin with the cells and ZOs maintains TJ integrity and controls the transport of the particles through the paracellular space [39]. Molecular structure of the intracellular junction of intestinal epithelial cells is shown in Figure 1. 

Intestinal barrier damage, i.e., compromised functioning of the intercellular junctions in the intestinal wall, results in a complete or partial loss of control over the agents that penetrate the bloodstream [40].

The TJs are structures that are responsible for the integrity and selectivity of the intestinal epithelium. They are also necessary to maintain balance between particular parts of the body, as well as between the body and the outer environment. For many years, research has investigated the factors that may be involved in controlling the functions of the intercellular junctions of the intestinal wall. Consequently, the effects of tumor necrosis factor α (TNF-α) and interferon gamma (IFN-γ) on TJ functioning have been confirmed. Moreover, researchers have demonstrated that myosin light chain kinase (MLCK) is involved in intestinal barrier regulation with TNF-α. In addition, research has indicated that MLCK activation itself reduces TJ permeability [41], while IFN-γ increases intestinal permeability through changes in the expression, distribution and location of the TJ proteins. IFN-γ is also responsible for cytoskeleton regrouping [42].

The pattern recognition receptors (PRRs), or pathogen recognition receptors, are key factors in the early innate immune response of the intestine. Toll-like receptors (TLRs) belong to the class of transmembrane PRRs that are important for the recognition of pathogens and the coordination of the immune response (TLR2, which identifies the patterns of both Gram-negative and Gram-positive bacteria, is particularly important here). In vitro TLR2 stimulation results in the activation of protein kinase C (PKC) and the movement/translocation of ZO-1 to the TJ complex. This ZO-1 location change is controlled by the PI3/Akt (phosphatidylinositide 3-kinase and the Akt protein kinase) signaling pathway, depending on the MYD88 (myeloid differentiation primary response 88) gene [43].

The proteinase-activated receptors (PARs) belong to the subfamily of the G-protein-coupled receptors that are activated by N-terminal proteolytic cleavage. The PAR2s are located on the apical and basolateral sides of the enterocytes. Activation of the basolateral PAR2s leads to increased intestinal permeability, resulting from separation of the transmembrane proteins, including ZO-1, occludin and actin [44].

Based on the literature data, it is known that claudins are essential components of the tight junctions that are responsible for intestinal barrier integrity [37]. Alterations in the structure of these tight junctions (i.e., decreased expression of certain proteins including claudin-3, claudin-4, claudin-5 and claudin-8) weakens the intercellular junctions and promotes the development of certain diseases (such as inflammatory bowel disease) [45]. Similar observations concerning the decreased expression of claudin-3 and claudin-4 have been made among children with celiac disease [46].

Moreover, Al-Sadi R et al. have demonstrated in their animal studies that occludins are necessary for the inhibition of the intercellular permeability of macroparticles [47]. In a properly functioning epithelial cell layer, occludin is strongly phosphorylated on its serine and threonine residues, while tyrosine phosphorylation is reduced to a minimum. In contrast, during the disruption of tight junctions, occludin undergoes dephosphorylation on its serine and threonine residues, and increased phosphorylation on its tyrosine residues [48]. Poor expression of this protein has been observed in patients with coeliac disease or irritable bowel syndrome [46,49].

There is a hypothesis that links increased intestinal permeability to the development of anti-Saccharomyces cerevisiae antibodies (ASCAs), which are present in both Crohn’s disease and celiac disease [50,51]. In Crohn’s disease, ASCAs appear to be a stable marker, whereas in patients with celiac disease, it has been reported that the incidence of ASCA IgA decreases after the introduction of a gluten-free diet (GFD). Studies also demonstrate that ASCA can be detected in a significant proportion of untreated celiac patients, regardless of the degree of mucosal damage [50].

## 4. Laboratory Diagnosis of the Intestinal Barrier Permeability Disorders

As the intestinal barrier is a very complex structure containing many components, it is difficult to assess its integrity. Various markers are sought to evaluate the intestinal permeability among patients with atopic diseases. The current tool is the lactulose:mannitol (L/M) test which is considered a non-invasive marker for the integrity and permeability of intestinal mucosa [52,53,54,55,56]. Other tests are also performed to assess the intestinal barrier, including tests with intestinal permeability markers such as zonulin and bacterial lipopolysaccharides (LPS), and tests that may indicate inflammation, therefore indirectly indicating increased intestinal permeability, such as the assessment of alpha-1-anti-trypsin levels [57,58,59].

## 5. Allergic Diseases

Over the last 20–30 years, a significant increase in the incidence of atopic diseases, i.e., asthma, allergic rhinitis and food allergies, has been observed worldwide. Studies available in the literature confirm that in the general population, the percentage of patients with atopic diseases is as high as 40%, and it still increasing [1,60]. Atopy is a hereditary predisposition to an abnormal immunological response to environmental factors that are neutral for the general population and is manifested by the excessive production of substance-specific IgE antibodies. On the other hand, an allergy is a specific, unfavorable reaction for the system that depends on the secondary immunological response to contact with an antigen [1,60,61].

Studies show that the most common clinical manifestations of allergies observed in children under three years of age are food allergies, with gastrointestinal and/or skin complaints. As a result, allergic diseases are an increasingly frequent reason for parents of infants and young children to contact their family physician, pediatrician or specialists in pediatric gastroenterology and allergology [1,2]. Environmental factors, such as excessive hygiene, air pollution, widespread use of antibiotics, changes in dietary habits, small families, an increase in caesarean-section births and urbanization are considered to be causes of the increased incidence of allergies [1,2,60].

According to current literature data, food allergy symptoms are present in over 5% of the entire adult population, and in nearly 8% of all children. The most common allergens include foods from the “big eight” group, i.e., cow’s milk, hen’s eggs, soybean, wheat, peanuts, other nuts (hazelnuts, walnuts), fish and crustaceans [61,62,63]. Importantly, literature data confirm that the main allergens causing clinical manifestations in the pediatric population are cow’s milk protein (2–3% of the entire study population) and egg white (2–2.5% of the entire study population) [61,64]. These products, especially milk, constitute an important part of the diet during early childhood and are essential for proper development.

Unfortunately, the only effective method for treating a food allergy is to eliminate the ingredient responsible for the development of disease symptoms from the child’s diet and to introduce ingredients with equivalent nutritional properties [65]. The aim of the elimination diet is primarily to calm the allergic reaction. This leads to the regeneration of gastrointestinal mucosa and, as a result, the improvement of digestive–absorptive function in the intestines, the reduction of excessive absorption through the mucosal barrier of protein antigens from the gastrointestinal lumen and the achievement of food tolerance. Effective treatment alleviates the disease symptoms until they completely disappear, which consequently improves the patient’s general condition and enables proper physical development [65,66]. In everyday practice, dietary treatment can be extremely difficult, especially when two or more products are eliminated from the child’s diet, or when elimination diets are used for a longer period of time. The resulting quantitative and qualitative restrictions in the composition of macronutrients and micronutrients may then be significant, and may result in impaired growth and maturation processes [65,66,67,68].

## 6. Pathomechanisms of Atopic Diseases and the Intestinal Barrier

The pathomechanism of atopic disease is significantly associated with an immature intestinal barrier, which is a subject of current research. The human intestinal barrier develops gradually during fetal development. Research conducted at the beginning of the twenty-first century has demonstrated an increase in the intestinal permeability in premature neonates and infants [69,70]. Such research reveals that the process of intestinal barrier maturation begins at approximately 38 weeks of gestation and continues after birth during the neonatal and infant periods. A premature infant is particularly vulnerable to protein antigens that penetrate the intestinal barrier, which may promote the development of allergies, especially in genetically predisposed infants [71].

**Figure 2:**
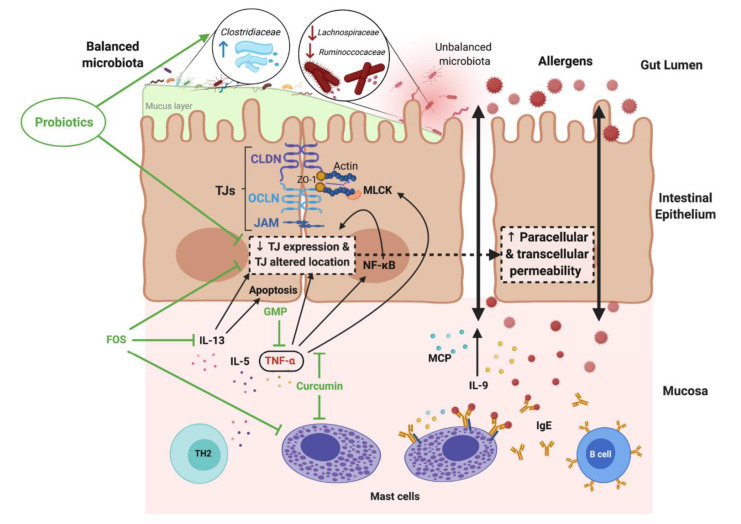
Increased intestinal permeability in food allergy (FA) and the modulatory effects of probiotic, prebiotic and food components. Reprinted with permission from Ref. [77]. 2021, Salinas, E. IL- interleukin; TNF-α- tumor necrosis factor α; CLDN- claudins; OCLN- occludins; GMP- glycomacropeptide; FOS- fructo-oligosaccharides; TH2- T-helper 2 cytokines; MPC- monocyte chemoattractant protein.

Several studies have linked increased intestinal barrier permeability to food allergies [72,73]. Research has shown that patients with atopic diseases respond abnormally to food allergens [74]. Accordingly, differences in the types of immune complexes formed in response to antigen challenges are significant, which seems to underlie the systemic signs of food allergies [75]. Increased intestinal permeability over the course of a food allergy allows allergens to penetrate through the intestinal barrier and stimulate the submucosal immune system (Figure 2). The release of cytokines and inflammatory mediators further enhances degradation of the epithelial barrier and leads to an improper cycle, resulting in increased intestinal permeability [76,77].

Several studies have also demonstrated increased permeability of the epithelial cells in those afflicted with atopic eczema and bronchial asthma [78,79].

Certain Italian studies demonstrate that the prevalence of celiac disease in atopic disease is significantly higher than in the general population, and patients with celiac disease present a significant overexpression of mucosal immunoreactivity [80]. Other Italian researchers have shown that in a group of more than 1000 patients with celiac disease (silent or latent form), atopy is the second most common concomitant disease, after insulin-dependent diabetes [81]. Therefore, atopy should be considered a risk factor, and patients with atopic disease should be routinely screened for celiac disease using specific antibodies (IgA EmA or IgA anti-tTG) [80]. The role of intestinal permeability markers among children with food allergies has not been fully understood, and the identification of biological indicators/markers that predict growth retardation in children with allergic diseases and atopy has not been well explained.

There are reports concerning the compromised functions of the tight junctions that lead to excessive intestinal permeability. These reports underline the role of tight junctions in the pathogeneses of several acute or chronic diseases in the pediatric population. This is probably initiated during infancy. Liu at al. provide evidence of tight junction degradation in such diseases as systemic inflammatory response syndrome (SIRS), inflammatory bowel disease, type 1 diabetes, bronchial asthma and autism [82].

In the population of patients with food allergies, the most common assessment of intestinal permeability is based on the lactulose:mannitol test. There are no available Polish or foreign tests that evaluate the usefulness of other markers, e.g., the bacterial lipopolysaccharide (LPS) assay in children with atopic diseases. However, one zonulin assay in this group of children has been found. The analyses performed by Sheen et al., who evaluated the role of circulating zonulin in AD development and severity, demonstrated that serum zonulin concentration was considerably higher among children with atopic diseases compared to the control group [83].

Studies conducted between 1994 and 2015 that assessed IP levels based on the L/M test in children and adults with AD have shown increased IP values in this patient group [52,53,54]. In addition, research by Järvinen et al. demonstrated increased permeability in approximately 40% of the study subjects with food allergies who were aged over six years [52]. Similarly, Laudat et al. also assessed intestinal barrier function, but among infants and young children with food allergies aged 2.3 ± 1.6 years, and they revealed increased intestinal barrier permeability in this group [53].

The available literature data concerning the evaluation of intestinal permeability in children with food allergies using the sugar-absorption test confirm the increased intestinal permeability in patients with these conditions. However, this research methodology may reduce interest in the L/M test and intensify the search for new methods to assess the intestinal barrier. The sugar-absorption test is time-consuming, is not standardized and does not have reference values [55]. Although these tests seem to be sensitive and useful for intestinal barrier assessment and for diagnosis, markers that do not burden patients and markers that are safe for their health are required. Perhaps zonulin and LPS will appear to be good IP markers.

Considering the existing relation between an abnormally functioning intestinal barrier and the pathogeneses of allergic, autoimmune, neurological or other diseases, access to intestinal permeability assessment methods, particularly in the pediatric population, seems to be key for the determination of the risk of disease in the future and even the severity of the pathological process. The identification of at-risk patients would enable preventive and diagnostic action, while finding the location of intestinal barrier damage might be the starting point for appropriate and personalized therapy or probiotic supplementation. While research on the modulation of intestinal permeability is still in the initial phase, the results are promising. Thus, it can be said that an increase in intestinal permeability is associated with abnormal intestinal mucosa, resulting in compromised nutrient absorption and digestion, which may lead to an increase in the risk of growth retardation and malnutrition.

## Data Availability

Not applicable.
